# Promoter methylation of transient receptor potential melastatin-related 7 (*TRPM7*) predicts a better prognosis in patients with Luminal A breast cancers

**DOI:** 10.1186/s12885-022-10038-z

**Published:** 2022-09-05

**Authors:** Yuanyuan Wang, Rong Lu, Pu Chen, Rongrong Cui, Meiju Ji, Xiaozhi Zhang, Peng Hou, Yiping Qu

**Affiliations:** 1 Department of Endocrinology, Key Laboratory for Tumor Precision Medicine of Shaanxi Province, The First Affiliated Hospital of Xi’an Jiaotong University, Xi’an, 710061 P.R. China; 2 Center for Translational Medicine, The First Affiliated Hospital of Xi’an Jiaotong University, Xi’an, 710061 P.R. China; 3 Department of Radiation Oncology, The First Affiliated Hospital of Xi’an Jiaotong University, Xi’an, 710061 P.R. China

**Keywords:** Breast cancer, *TRPM7*, Promotor methylation, Methylation-Specific PCR (MSP), Clinical outcomes

## Abstract

Breast cancer is the most common female tumors arising worldwide, and genetic and epigenetic events are constantly accumulated in breast tumorigenesis. The melastatin-related transient receptor potential 7 channel (*TRPM7*) is a nonselective cation channel, mainly maintaining Zn^2+^, Ca^2+^ and Mg^2+^ homeostasis. It is also involved in regulating proliferation and migration in various cancers including breast cancer. However, epigenetic alterations (such as promoter methylation) of *TRPM7* and their correlation with clinical outcomes in breast cancer patients remain largely unclear. In this study, we found that *TRPM7* was highly expressed in the luminal A subtype of breast cancers but no other subtypes compared with GTEx (Genotype-Tissue Expression Rad) or normal samples by analyzing the TCGA database. Correspondingly, *TRPM7* was methylated in 42.7% (93 of 219) of breast cancers. Further studies found that promoter methylation of *TRPM7* were significantly associated with better clinical outcomes in breast cancer patients, especially in the Luminal A subtype. Besides, methylated *TRPM7* was correlated with less number of metastatic lymph nodes and longer local failure free survival time in this subtype. In summary, our data indicate that promoter methylation of *TRPM7* may predict poor prognosis in patients with luminal A breast cancer.

**Supplementary Information:**

The online version contains supplementary material available at 10.1186/s12885-022-10038-z.

## Introduction

Breast cancer (BC) is one of the leading cause cancers that affect women health all around the world [1].It is thus vital to gain a better understanding of the molecular mechanisms underlying the development of this disease [2]. In recent years, many researchers have been devoted to find and validate molecular alterations to serve as a prognostic and predictive biomarker. One of the validated and widely used multi-gene signature tests is the 21 genes, which is commonly applied for predicting the breast cancer outcomes [3, 4]. In addition, there are also studies demonstrating that epigenetic alterations, such as promoter hypermethylation or hypomethylation, can lead to aberrant gene expression in tumor cells [5, 6]. What’s more, the extension and functional importance of metabolic alterations and the associated genes also become a hot topic study in numerous cancers.

It is the fact that mammary microcalcifications occur in 30% to 50% of breast cancer patients, which are frequently associated with poor patient survival [7, 8]. Moreover, serum calcium (Ca^2+^) has been proved to be associated with the risk of breast cancer (9). Besides, calcium signals play important roles in tumorigenesis by interacting with and modulating tumor microenvironment [9, 10]. In breast cancer cells, intracellular Ca^2+^ is maintained via two classes of Ca2 + channels [11]. The transient receptor potential (TRP) super family of ion channels contains about thirty members, forming a non-selective cation permeable channels and is organized into seven subgroups according to their sequence homology [12, 13]. The transient receptor potential cation channel melastatin-subfamily (TRPM), composed of eight members from TRPM1 to TRPM8, is involved in various physiological functions including carcinogenesis [12].

The transient receptor potential cation channel melastatin-subfamily, member 7 (TRPM7, ChaK1, TRP-PLIK andLTRPC7), is ubiquitously expressed in many tissues [14, 15]. This protein is made up of 6 transmembrane domains and a COOH-terminal α-kinase domain [16]. The TRPM7 ion channel is involved in various physiological and pharmacological processes both with its channel activity and its kinase activity [15, 17]. The crucial role of TRPM7 in the balance of Zn(2 +), Mg(2 +), Ca(2 +) implicated in many metabolic processes and signalling pathways [18–21]. Through its varied physiological roles, TRPM7 is of major importance in much human pathology in various cancers, including breast cancer [22–24]. For example, in prostate cancer, the upregulation of TRPM7 enhanced the migration and invasion potential of cancer cells by promoting epithelial–mesenchymal transition (EMT) process [25, 26]. Similarly, knocking down TRPM7 in pancreatic cancer cells inhibited cancer cell migration [27]. For gastric cancer, several studies suggest that TRPM7 participates in the survival of human gastric adenocarcinoma cells by acting as detoxifiers [28–30].Also, there are studies suggesting that TRPM7 expression is significantly higher in invasive breast ductal carcinomas compared with control subjects, and demonstrating that TRPM7 is involve in regulating breast cancer cell proliferation, apoptosis, EMT, migration, invasion and microcalcification [31, 32]. Given that approximately 15%-20% of breast cancer patients diagnosed with triple negative breast cancer (TNBC) in the United States and lack appropriate targeted therapeutic drugs, chemical modulators of the TRPM7 channel might potentially be used in therapeutic applications [33, 34].

Considering that most previous studies focus on the expression of TRPM7 and its role in promoting malignant transformation of numerous cancers, the aims of the present study are to investigate the methylation status of *TRPM7* in a cohort of breast cancer patients and its association with their clinicopathological features, better understanding the pathogenesis of breast cancer.

## Patients and methods

### Patients

With the approval of our institutional review board and human ethics committee, where required, a total of 219 paraffin-embedded breast cancer tissues were randomly obtained at the First Affiliated Hospital of Xi’an Jiaotong University. All individuals signed informed consent to participate in the study and familial cases of breast cancer were excluded. Tumor staging was performed according to the tumor, node, and metastasis (TNM) classification. In addition, patients who showed the breast and/or ovarian cancers in the first- and second-degree relatives were excluded from the study. Tumor samples were obtained following surgical resection and their pathological features were examined on macro dissection of the samples. All samples were histologically examined by a senior pathologist at Department of Pathology of the Hospital based on World Health Organization (WHO) criteria. The demographic and clinical features of the study population are summarized in Table 1.

**Table 1:** Clinicopathological characteristics of breast cancer patients (*n* = 219)

Characteristics	Percentage of Patients (n/n)	Methylation frequency of *TRPM7*(n/n)
Age,years		
Mean	51.5	n/a
SD	11.5	n/a
WHO grade		
I	3.2(7/219)	28.6(2/7)
II	74.4(163/219)	45.4(74/163)
III	22.4(49/219)	34.7(17/49)
Molecular Subtype		
Luminal A	35.6(78/219)	33.3(26/78)
Luminal B	32.0(70/219)	45.7(32/70)
Her2^+^	14.6(32/219)	46.9(15/32)
Basal like	17.8(39/219)	51.3(20/39)
ER		
-	37.0(81/219)	37.0(30/81)
+	63.0 (138/219)	45.6(63/138)
PR		
-	43.8(96/219)	38.5(37/96)
+	56.2(123/219)	45.5(56/123)
Her2		
-	78.1(171/219)	45.0(77/171)
+	21.9(48/219)	33.3(16/48)
Lymph node metastasis (LNM)		
No	69.8(153/219)	56.2(86/153)
Yes	30.2(66/219)	10.6(7/66)*
Radiotherapy		
No	51.6(113/219)	54.0(61/113)
Yes	48.4(106/219)	30.2(32/106)
Chemotherapy		
No	14.2(31/219)	54.8(17/31)
Yes	85.8(188/219)	40.4(76/188)
Recurrence		
No	84.9(186/219)	44.6(83/186)
Yes	15.1(33/219)	30.3(10/33) *
Survival status		
Alive	82.6(181/219)	45.3(82/181)
Dead	17.4(38/219)	28.9(11/38)*

*stand for *p* <0.05

### DNA extraction and bisulfite modification

As shown in our previous study [35]. All tissues sections were reviewed by board certified pathologists to ensure that ≥ 50% of the cells used for DNA purification were neoplastic. The tissues were first treated with xylene for 12 h at room temperature to remove the paraffin, and were then subjected to digestion with 1% sodium dodecylsulfate (SDS) and proteinase K at 48 °C for 48 to 72 h with the addition of several spiking aliquots of concentrated proteinase K to facilitate digestion. Next, genomic DNA was isolated from the digested tissues followed by a standard phenol–chloroform extraction and ethanol precipitation protocol. The samples were then stored at -80 °C until use. Quantity and quality of the extracted DNA were evaluated by a spectrophotometer (Nanodrop 2000, Thermo Scientific, USA). About 1 μg of genomic DNA was then treated with bisulfite to convert unmethylated cytosine to uracil prior to a methylation-specific PCR (MSP) using Epi Tect Bisulfite Kit (Qiagen, Germany) according to the manufacturer’s instructions.

### MSP assay

The bisulfite-modified DNA was amplified using specific methylated or unmethylated primersas follow: 5’-TCGGTATAGGTTAGGTTTAGTTAGC-3’ (forward) and 5’-AAAATAATAAAATATTCACGCCGTA-3’ (reverse) for methylated gene; 5’-TGGTATAGGTTAGGTTTAGTTAGTGG-3’ (forward) and 5’-AAAATAATAAAATATTCACACCATA-3’ (reverse) for unmethylated gene. As shown in our previous study [35]. PCR amplification was then carried out in the buffer containing 16.6 mM ammonium sulfate, 67 mM Tris base, 2.5 mM MgCl2, 10 mM 2-mercaptoethanol, 0.1% DMSO, 0.2 mM each of dATP, dCTP, dGTP and dTTP, 600 nM each of forward and reverse primers and 0.6 unit Platinum Taq polymerase. Each sample was run in triplicate. The condition for *TRPM7* amplification was 10 min at 95 °C, followed by amplification cycles including 30 s at 95 °C, 30 secs at 60 °C and 30 s at 72 °C for the extension, in addition to final elongation of 10 min at 72 °C. Normal leukocyte DNA was methylated in vitro with *Sss I* methylase (New England Biolabs, Beverly, MA) to generate completely methylated DNA as a positive control. After PCR, electrophoresis was performed on a 1.5% agarose gel.

### Statistical analysis

Data were analyzed using SPSS 16.0 (SPSS Inc. Chicago, USA). As shown in our previous study [35].The Mann–Whitney *U* test and Kruskal–Wallis test were performed for numerical data and the Chi-square test was used to analyze the relationship between parameter data. Multivariate models were then developed that adjusted for the most important covariates, including age, tumor size, differentiation and lymph node metastasis. Survival length was determined from the day of primary tumor surgery to the day of death or last clinical follow-up. The Kaplan–Meier method was used for survival analysis grouping with methylation status of *TRPM7*. Differences between curves were analyzed using the log-rank test. Multivariate Cox regression analysis was used to evaluate the effect of gene methylation on survival of independently of the number of lymph node metastasis, tumor invasion and differentiation. Differences were considered statistically significant if *P* < 0.05.

## Results

### Different expression of *TRPM7* is correlated with molecular subtype in breast cancers

**Fig. 1:**
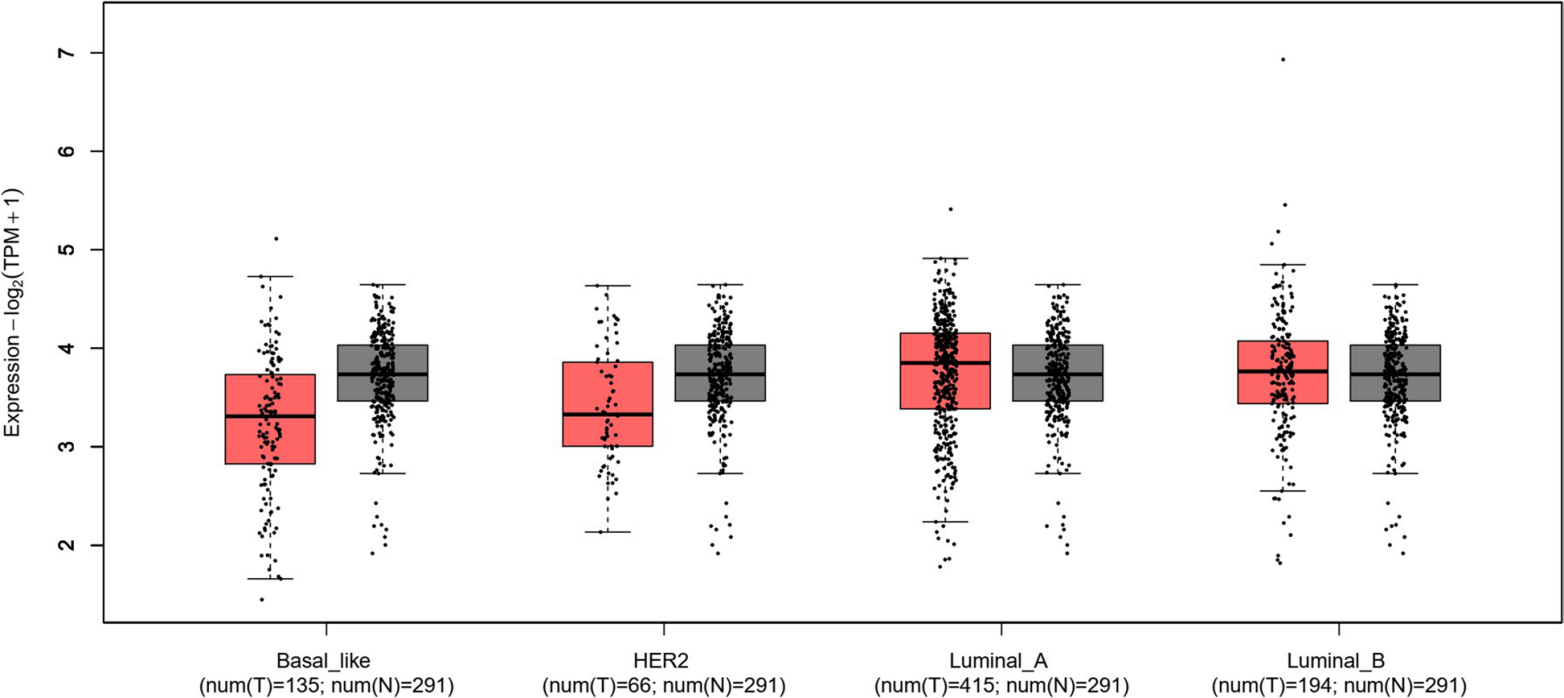
Expression of *TRPM7* in breast cancer and normal tissues. In TCGA database, compared with GTEx (Genotype-Tissue Expression Rad), the expression of *TRPM7* was lower in Basal like and Her2 + breast cancers. However, the expression of *TRPM7* was higher in the Lumina A and Luminal B subtypes

**Fig. 2:**
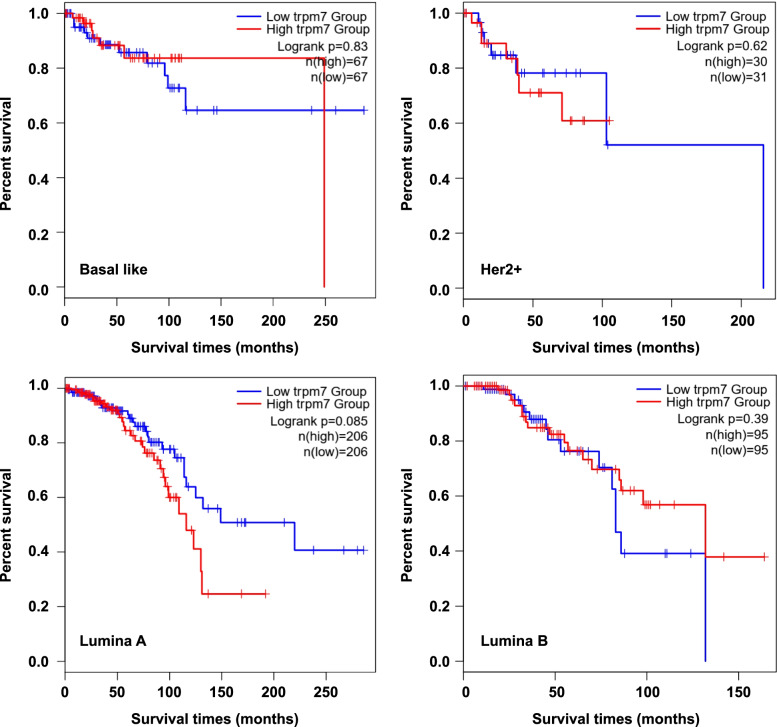
High level of *TRPM7* leads to poor prognosis of breast cancer patients. Expression of *TRPM7* gene causes shorter survival times after surgery especially in the Lumina A subtype

Previous studies have indicated that high level of TRPM7 proteins is correlated with shorter survival time in breast cancer after surgery. By analyzing mRNA level of *TRPM7* in TCGA database using the online tools GEPIA (http://gepia2.cancer-pku.cn) (Fig. 1), we found that the expression of *TRPM7* differed from the different subtype of breast cancer tissues. Although not significantly, the expression of *TRPM7* in the Lumina A and Luminal B subtypes was higher, and was lower in the Basal-like and Her2 positive subtypes than either GTEx or normal samples (data from TCGA database). Next, we also found that high expression of *TRPM7* was related to poor patient survival in the Lumina A subtype, although it did not reach statistical significance (Fig. 2). These observations, taken together, support that TRPM7 might act as an oncogene, and suggest that it may be a predictor for poor survival in patients with Lumina A breast cancers.

### Association of promoter methylation of *TRPM7* with clinicopathological features in patients with breast cancers

Considering that the regulatory effect of promoter methylation on gene expression in human cancers [27, 28], we next examined promoter methylation of *TRPM7* using MSP approach in a cohort of breast cancers and control subjects. As shown in Table 1, the methylated rate of *TRPM7* was 42.7% (93/219) in the whole cohort. In consonance with TCGA database shown high expression of TRPM7 in the Lumina A subtype, we found the methylation frequency of *TRPM7* was much lower in Lumina A subtype (33.3%) than that in others subtypes (51.3% in Basal-like, 46.9% in Her2^+^, and 45.7% in Luminal B). Moreover, we also found methylation frequency of *TRPM7* was negatively correlated with lymph node metastasis, disease recurrence and cancer related death. However, no significant difference was seen between *TRPM7* methylation with TNM, therapy strategies and hormone receptors.

We also examined the relationship of *TRPM7* methylation status with clinicopathological characteristics using logistic regression analysis. The univariate analyses showed that *TRPM7* methylation status was significantly associated with molecular subtypes (OR = 1.27; 95% CI = 1.00–1.63; *P* = 0.05), lymph node metastasis (OR = 0.09; 95% CI = 0.04–0.22; *P* = 0.001), and radiotherapy (OR = 0.37; 95% CI = 0.21–0.64; *P* = 0.001) (Table 2). Besides, although the difference did not reach statistical significance, the cancer related death also trended to be associated with *TRPM7* methylation (OR = 0.49; 95% CI = 0.23–1.05; *P* = 0.06). In order to assess the independent association of gene methylation with molecular subtypes (including Basal like, Her2 positive, Luminal A and Luminal B), lymph node metastasis, endocrine therapy, radiotherapy, chemotherapy, relapse and cancer-related death, we conducted multiple multivariable logistic regression analysis. Also shown in Table 2 and Figure S1, *TRPM7* methylation remained negatively associated with lymph node metastasis (OR = 0.09; 95% CI = 0.03–0.27; *P* = 0.001) and cancer related death (OR = 0.20; 95% CI = 0.03–1.21; *p* = 0.07).

**Table 2:** *TRPM7*methylation in breast cancer: univariate and multivariate models with clinicopathological characteristics

Characteristics	Univariate		Multivariate	
	OR^a^ (95% CI)	*P*	OR^a^ (95% CI)	*P*
Age^b^	1.04 (0.84–1.28)	0.75	/	/
WHO grade^c^	0.66 (0.37–1.18)	0.16	/	/
Molecular subtype^d^	1.27 (1.00–1.63)	0.05	1.34 (0.94–1.90)	0.10
LNM^e^	0.09 (0.04–0.22)	0.001	0.09 (0.03–0.27)	0.001
ER	1.43 (0.81–2.50)	0.21		
PR	1.33 (0.77–2.29)	0.30		
Her2	0.61 (0.31–1.20)	0.15		
Endocrine therapy	1.43 (0.81–2.50)	0.21	1.45 (0.76–2.76)	0.26
Radiotherapy	0.37 (0.21–0.64)	0.001	0.94 (0.36–2.44)	0.90
Chemotherapy	0.56 (0.26–1.20)	0.14	0.09 (0.31–1.97)	0.61
Relapse	0.63 (0.29–1.38)	0.25	3.25 (0.49–21.58)	0.22
Survival status^f^	0.49 (0.23–1.05)	0.06	0.20 (0.03–1.21)	0.07

^a^OR: odds ratio with 95% confidence interval; ^b^Age (per 10 years); ^c^WHO grade (I, II, III and IV); ^d^Molecular subtype (Luminal A, Luminal B, Her2 positive and Basal like); ^e^Lymph node metastasis; ^f^Survival status (alive vs. dead)

Next, we further examined the relationship of *TRPM7* methylation with clinicopathological characteristics in patients with different molecular subtypes using logistic regression. As shown in Table 3, *TRPM7* methylation was negatively associated with lymph node metastasis and radiotherapy in patients with Her2 positive (Figure S2) and Lumina A subtypes (Figure S3). In the Lumina B subtype (Figure S4), we similarly found that *TRPM7* methylation was negatively associated with lymph node metastasis and radiotherapy, while positively associated with endocrine therapy. In the Basal like subtype, we failed to find the above relationships. Notably, our analysis showed that TRPM7 methylation trended to have a negative correlation with cancer-related death only in the Lumina A subtype (Table 3 and Figure S3).We then conducted multiple multivariable logistic regression analysis in Lumina A and Lumina B subtypes, and expectedly found that *TRPM7*methylationwas negatively associated with lymph node metastasis in Lumina A subtype, while positively associated with endocrine therapy in Lumina B subtype (Table 4).These findings further support the above conclusions.

**Table 3:** Univariate analysis of *TRPM7* methylation with clinicopathological characteristics in different subtypes of breast cancers

Characteristics	Basal like		Her2 positive		Luminal A		Luminal B	
	OR^a^ (95% CI)	*P*	OR^a^ (95% CI)	*P*	OR^a^ (95% CI)	*P*	OR^a^ (95% CI)	*P*
Age^b^	0.98 (0.61–1.58)	0.93	0.78 (0.42–1.46)	0.44	0.80 (0.54–1.21)	0.30	1.41 (0.98–2.05)	0.07
WHO grade^c^	0.59 (0.15–2.34)	0.45	0.32 (0.07–1.44)	0.14	1.19 (0.44–3.27)	0.73	0.47 (0.15–1.45)	0.19
LNM^d^	/	/	0.08 (0.009–0.76)	0.03	0.06 (0.007–0.47)	0.008	0.19 (0.06–0.58)	0.004
ER	/	/			0.57 (0.22–1.49)	0.25	4.82 (1.62–14.36)	0.005
PR	/	/			0.53 (0.20–1.38)	0.19	3.51 (1.29–9.59)	0.01
Her2	/	/	1.87 (0.27–13.09)	0.53	0.74 (0.25–2.21)	0.59	0.46 (0.13–1.67)	0.24
Endocrine therapy	/	/	/	/	0.57 (0.22–1.49)	0.25	4.82 (1.62–14.36)	0.005
Radiotherapy	/	/	0.05 (0.005–0.47)	0.009	0.25 (0.08–0.82)	0.02	0.18 (0.06–0.53)	0.002
Chemotherapy	0.32 (0.03–3.33)	0.34	0.88 (0.05–15.33)	0.93	0.57 (0.18–1.75)	0.33	0.37 (0.09–1.63)	0.19
Relapse	0.54 (0.13–2.34)	0.41	2.46 (0.20–30.28)	0.48	0.26 (0.03–2.21)	0.22	0.54 (0.15–1.98)	0.35
Survival status^4^	0.54 (0.13–2.34)	0.41	0.54 (0.04–6.58)	0.63	0.31 (0.06–1.52)	0.15	0.54 (0.15–1.45)	0.35

^a^OR: odds ratio with 95% confidence interval; ^b^Age (per 10 years); ^2^WHO grade (I, II, III and IV); ^c^Lymph node metastasis; ^d^Survival status (alive *vs*. dead)

**Table 4:** Multivariate analysis of *TRPM7* methylation with clinicopathological characteristics in Luminal A and B breast cancers

Characteristics	Luminal A		Luminal B	
	OR^a^ (95% CI)	*P*	OR^a^ (95% CI)	*P*
Age^b^	0.91 (0.58–1.43)	0.68	1.57 (0.95–2.59)	0.08
LNM^c^	0.03 (0.001–0.59)	0.02	0.55 (0.07–4.63)	0.59
Endocrine therapy	0.60 (0.19–1.83)	0.37	8.49 (2.10–34.29)	0.003
Radiotherapy	2.62 (0.22–31.59)	0.45	0.27 (0.03–2.20)	0.22
Chemotherapy	0.65 (0.18–2.38)	0.51	2.65 (0.40–17.55)	0.31
Survival status^d^	0.26 (0.05–1.36)	0.11	0.56 (0.11–2.76)	0.48

^a^OR: odds ratio with 95% confidence interval; ^b^Age (per 10 years); ^c^Lymph node metastasis; ^d^Survival status (alive *vs*. dead)

### Promoter methylation of*TRPM7* predicts better prognosis in patient with Lumina A breast cancers

Whether *TRPM7* methylation predicts a better survival in patients with Lumina A breast cancers, as suggested by its association with clinicopathological characteristics of this subtype of breast cancer patients, was subsequently investigated by univariate and multivariable survival analysis. As shown in Table 5, both univariate (Figure S5) and multivariate Cox regression showed that *TRPM7* methylation was a potential predictor of better survival for the whole cohort breast cancer patients. Next, we analyze the methylated status of *TRPM7* in the different molecular subtypes. As shown in Table 6, *TRPM7* methylation was only associated with better prognosis in the Lumina A subtype. Cox multivariate regression showed that *TRPM7* methylation (HR = 0.13, 95% CI = 0.02–0.72, *P* = 0.02) and endocrine therapy (HR = 0.03, 95% CI = 1.10–1.06, *P* = 0.06) is a predictor of better survival in Lumina A patients as an independently variable with respect to the age, LNM, radiotherapy and chemotherapy (Table 7 and Figure S6).

**Table 5:** Prognostic value of clinicopathological factors and *TRPM7* methylation using univariate and multivariate Cox regression analysis

Characteristics	Univariate		Multivariate	
	HR^a^ (95% CI)	*P*	HR^a^ (95% CI)	*P*
*TRPM7* methylation	0.50 (0.25–1.01)	0.06	0.50 (0.23–1.08)	0.07
Age^b^	1.10 (0.86–1.40)	0.45	1.15 (0.88–1.52)	0.31
WHO grade^c^	1.12 (0.56–2.23)	0.75	/	
Molecular subtype^d^	1.14 (0.86–1.53)	0.36	1.15 (0.81–1.63)	0.45
LNM^e^	1.28 (0.66–2.48)	0.46	0.91 (0.31–2.65)	0.86
Endocrine therapy	0.63 (0.33–1.18)	0.15	0.69 (0.36–1.32)	0.26
Radiotherapy	1.32 (0.69–2.51)	0.39	1.00 (0.34–2.97)	0.99
Chemotherapy	1.48 (0.53–4.19)	0.45	1.46 (0.46–4.64)	0.52
Relapse	83.01 (35.58–212.77)	0.001	/	

^a^HR: hazard ratio with 95% confidence interval (CI); ^b^Age (per 10 years); ^c^WHO grade (I, II, III and IV); ^d^Molecular subtypes (Luminal A, Luminal B, Her2 positive and Basal like); ^e^Lymph node metastasis

**Table 6:** Prognostic value of clinicopathological factors and *TRPM7* methylation using univariate Cox regression analysis in different subtypes of breast cancers

Characteristics	Basal like		Her2 positive		Luminal A		Luminal B	
	HR^a^ (95% CI)	*P*	HR^a^ (95% CI)	*P*	HR^a^ (95% CI)	*P*	HR^a^ (95% CI)	*P*
*TRPM7* methylation	0.69 (0.20–2.45)	0.57	0.53 (0.05–5.88)	0.61	0.26 (0.06–1.18)	0.08	0.58 (0.18–1.94)	0.38
Age^b^	1.20 (0.74–1.93)	0.46	1.56 (0.63–3.85)	0.34	0.98 (0.62–1.55)	0.94	1.04 (0.69–1.58)	0.85
WHO grade^c^	0.89 (0.21–3.79)	0.88	1.22 (0.13–11.47)	0.86	0.53 (0.14–2.05)	0.36	2.29 (0.73–7.20)	0.16
LNM^d^	2.63 (0.76–9.09)	0.13	4.65 (0.42–52.04)	0.21	0.65 (0.18–2.39)	0.52	0.88 (0.27–2.94)	0.84
Endocrine therapy	/	/	/	/	0.45 (0.15–1.33)	0.15	0.60 (0.19–1.87)	0.38
Radiotherapy	/	/	3.77 (0.34–41.71)	0.28	0.50 (0.14–1.83)	0.29	1.28 (0.41–3.98)	0.66
Chemotherapy	0.84 (0.11–6.68)	0.87	/	/	1.65 (0.36–7.48)	0.52	1.63 (0.21–12.61)	0.64

^a^HR: hazard ratio with 95% confidence interval (CI); ^b^Age (per 10 years); ^c^WHO grade (I, II, III and IV); ^d^Lymph node metastasis

**Table 7:** Prognostic value of clinicopathological factors and *TRPM7* methylation using multivariate Cox regression in Luminal A

Characteristics	HR^a^ (95% CI)	*P*
*TRPM7* methylation	0.13 (0.02–0.72)	0.02
Age^b^	1.04 (0.64–1.69)	0.89
LNM^c^	/	0.95
Endocrine therapy	0.33 (0.10–1.06)	0.06
Radiotherapy	/	0.94
Chemotherapy	1.84 (0.36–9.43)	0.46

^a^HR: hazard ratio with 95% confidence interval (CI); ^b^Age (per 10 years);^c^Lymph node metastasis

**Fig. 3:**
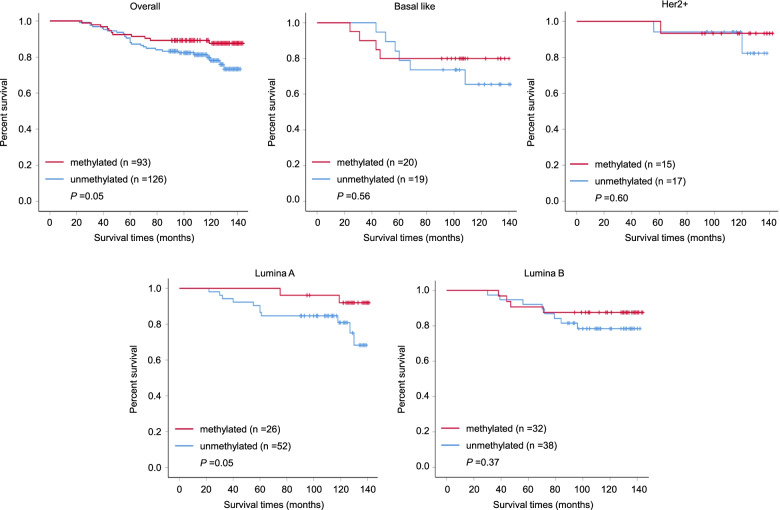
Methylation of *TRPM7* predicted longer survival time in Lumina A breast cancer patients. The Kaplan–Meier estimator of the survivorship function was used to evaluate the impact of aberrant methylation of *TRPM7* in the whole cohort breast cancer patients and the different molecular subtype cancers

The Kaplan–Meier estimator of the survivorship function is generally used to evaluate the impact of aberrant gene methylation on the survival of breast cancer patients. In the present study, we found that high expression of *TRPM7* trended to be associated with poor survival only in the Lumina A breast cancer patients by analyzing TCGA database (Fig. 2).This was also supported by our data showing a negative relationship between*TRPM7*methylation with survival times after surgery (median survival time: 133.3 *vs* 125.1 months; *P* = 0.05) in our cohort (Fig. 3). Further analysis showed that *TRPM7* methylation was negatively associated with poor survival only in patients with Lumina A subtype, but not in other subtypes, further supporting the above conclusion. Interestingly, *TRPM7* methylation also trended to predict longer local failure free survival time in this subtype (Fig. 3). Median time before disease recurrence was 129.2 *vs* 111.7 months in the whole cohort, while no disease recurrence was seen in the methylated *TRPM7* group in in the Lumina Asubtype. These results, taken together, suggest that *TRPM7* methylation may predict better prognosis in patients with luminal A breast cancers.

## Discussion

Cancer is still a leading cause of death worldwide and the incidence and mortality is growing rapidly due to the increased life expectancy and lifestyle issues [36].The complexity of breast cancer development and the recurrence of this disease spur the researchers for developing new targets for breast cancer diagnosis and treatment [37–39]. Previous studies have shown that *TRPM7* might be considered as a potential target for breast cancer treatment [29].

Numerous studies have shown that the TRP ion channels family genes now consists of more than 30 candidates, most of which are permeable for Zn^2+^, Ca^2+^, and some also for Mg^2+^ [18–22, 40, 41]. TRPM7 and TRPM8 has proved act as oncogenes in breast cancer tissues compared with normal tissues, and is correlated with the Scarff-Bloom-Richardson (SBR) grade, Ki67 and tumor size [31]. TRPM7 can also participate in cancer cell adhesion and migration via myosin-IIA filament and the MAPK signaling pathways. Besides, it can also affect protein localization by phosphorylating the heavy chain [32, 42].

Although the molecular function of TRPM7 in breast cancer cells has been widely studied, promoter methylation of *TRPM7* in breast cancers and its correlationship with clinicopathological characteristics of patients remains largely unclear. In the present study, we investigated promoter methylation of *TRPM7* in a cohort of breast cancers. Firstly, we analyzed the expression of TRPM7 using TCGA database, and found that its expression was higher in breast cancers than that in control subjects. In addition, we also observed that increased expression of *TRPM7* predict poor survival in Lumina A breast cancer patients. We then examined promoter methylation of *TRPM7* using MSP approach in a cohort of breast cancers and control subjects, and found the methylation frequency of *TRPM7* was 42.7% in the whole cohort. However, different molecular subtypes seem differs in the methylation rate. Next, we further examined the relationship of *TRPM7* methylation with clinicopathological characteristics, and found that *TRPM7*methylation was found to be significantly associated with molecular subtypes, lymph node metastasis, disease recurrence and cancer related death. In addition, univariate and multivariate Cox regression showed that *TRPM7* methylation served as a predictor of better survival in Lumina A breast cancer patients. The Kaplan–Meier estimator of the survivorship function also indicated that *TRPM7* methylation was negatively associated with longer survival time of breast cancer patients either after surgery or before disease recurrence.

## Conclusion

In conclusion, this is the first study which investigates the methylation status of *TRPM7* gene in breast cancers, providing the evidences for a possible pathogenic role of *TRPM7* in the development of breast cancer and suggesting that *TRPM7* methylation predicts a better patient prognosis in patients with Lumina A breast cancers. However, the limitation of our study is that we do not determine the reason why methylation frequency of *TRPM7* varies in different subtypes of breast cancer, and the relationship between *TRPM7* methylation and gene expression status.

## Supplementary Information

**Additional file 1: Figure S1.** Univariate analysis of *TRPM7* methylation with clinicopathological characteristics in breast cancers. *TRPM7* methylation negatively associated with lymph node metastasis and cancer related death.

**Additional file 2: Figure S2.** Univariate analysis of *TRPM7* methylation with clinicopathological characteristics in Her2 positive breast cancers. *TRPM7* methylation was negatively associated with lymph node metastasis and radiotherapy in patients with Her2 positive cancer patients.

**Additional file 3: Figure S3.** Univariate analysis of *TRPM7* methylation with clinicopathological characteristics in Luminal A breast cancers. *TRPM7* methylation was negatively associated with lymph node metastasis and radiotherapy in patients with Liminal A cancer patients.

**Additional file 4: Figure S4.** Univariate analysis of *TRPM7* methylation with clinicopathological characteristics in Luminal B breast cancers. *TRPM7* methylation was negatively associated with lymph node metastasis and radiotherapy, while positively associated with endocrine therapy in Luminal B breast cancers.

**Additional file 5: Figure S5.** Prognostic value of clinicopathological factors and *TRPM7* methylation using univariate Cox regression analysis in breast cancers. *TRPM7* methylation was a potential predictor of better survival for the whole cohort breast cancer patients.

**Additional file 6: Figure S6.** Prognostic value of clinicopathological factors and *TRPM7* methylation using multivariate Cox regression analysis in Luminal A breast cancers. Cox multivariate regression showed that *TRPM7* methylation and endocrine therapy is a predictor of better survival in Lumina A patients as an independently variable with respect to the age, LNM, radiotherapy and chemotherapy.

## Data Availability

All data generated or analyzed during this study are included in this article. Further enquiries can be directed to the corresponding author.
