# Supplementary figures and images for: Promoter methylation of transient receptor potential melastatin-related 7 (TRPM7) predicts a better prognosis in patients with Luminal A breast cancers
Source: BMC Cancer. 2022 Sep 5;22:951. doi: 10.1186/s12885-022-10038-z (PMC9446581; doi:10.1186/s12885-022-10038-z)

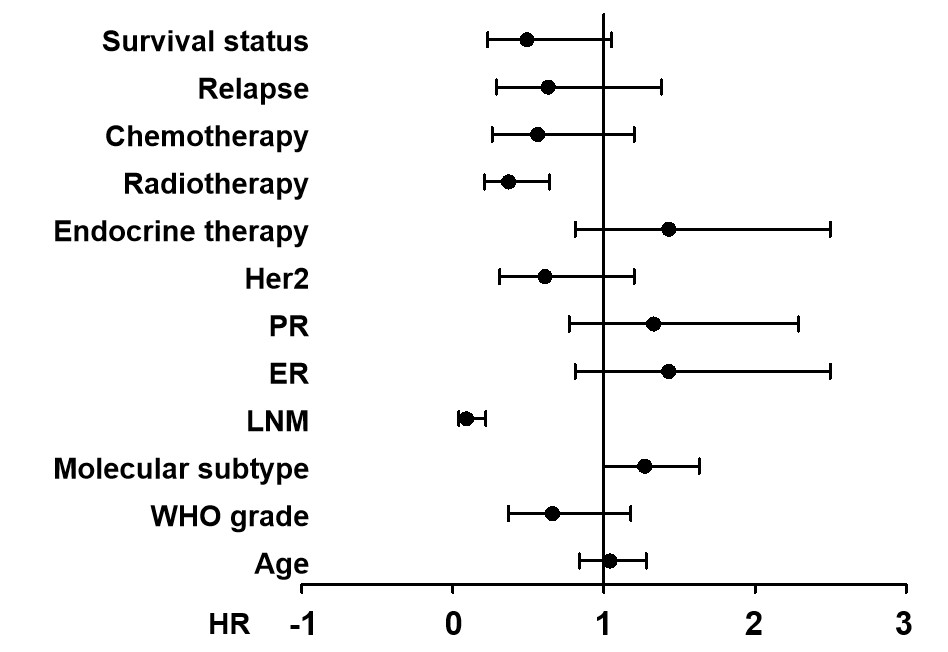

Supplement: Supplementary file 1 — Additional file 1: Figure S1. Univariate analysis of TRPM7 methylation with clinicopathological characteristics in breast cancers. TRPM7 methylation negatively associated with lymph node metastasis and cancer related death. [file 12885_2022_10038_MOESM1_ESM.jpg]

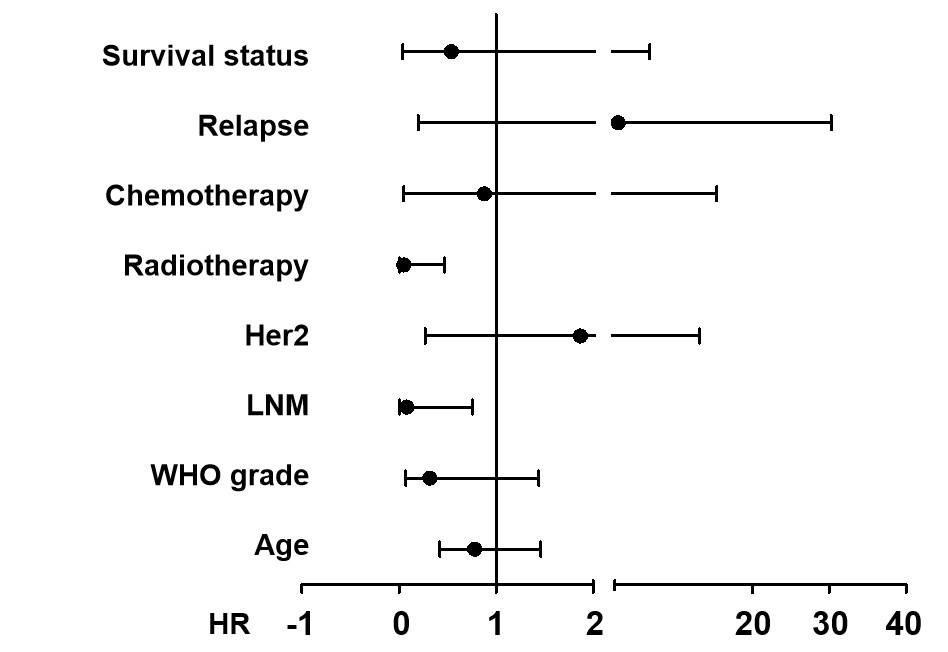

Supplement: Supplementary file 2 — Additional file 2: Figure S2. Univariate analysis of TRPM7 methylation with clinicopathological characteristics in Her2 positive breast cancers. TRPM7 methylation was negatively associated with lymph node metastasis and radiotherapy in patients with Her2 positive cancer patients. [file 12885_2022_10038_MOESM2_ESM.jpg]

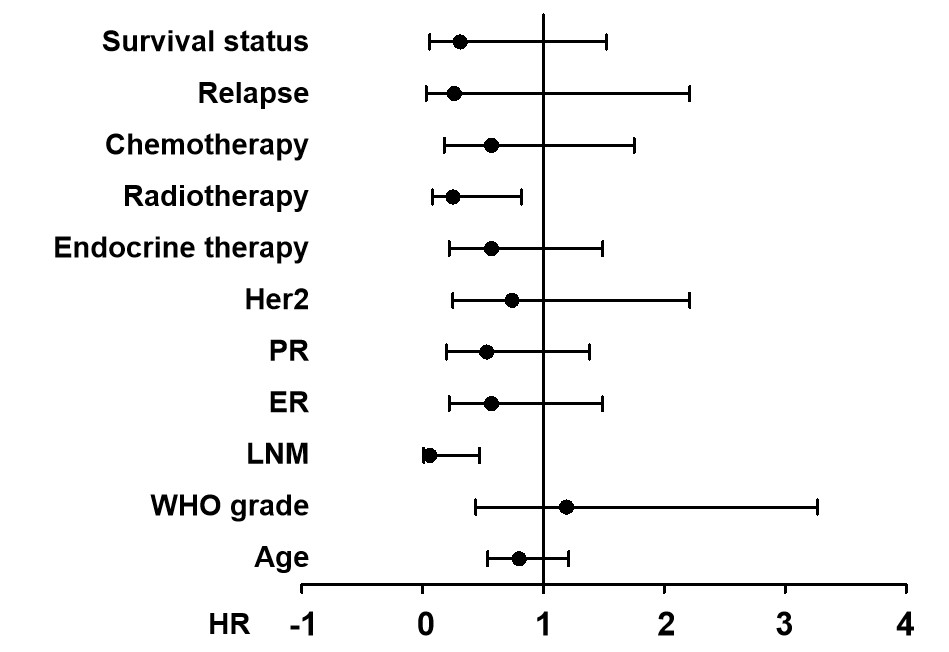

Supplement: Supplementary file 3 — Additional file 3: Figure S3. Univariate analysis of TRPM7 methylation with clinicopathological characteristics in Luminal A breast cancers. TRPM7 methylation was negatively associated with lymph node metastasis and radiotherapy in patients with Liminal A cancer patients. [file 12885_2022_10038_MOESM3_ESM.jpg]

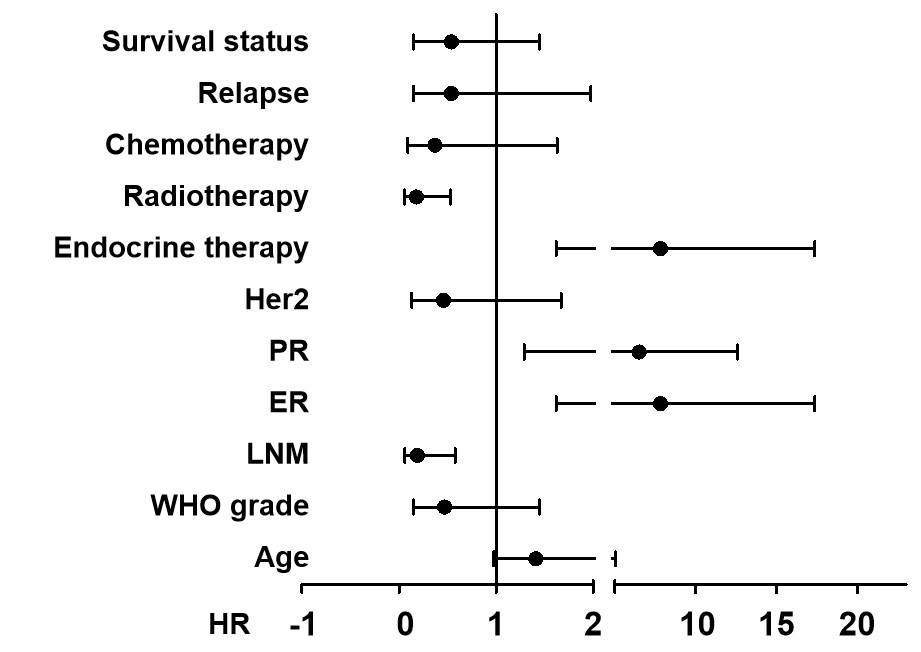

Supplement: Supplementary file 4 — Additional file 4: Figure S4. Univariate analysis of TRPM7 methylation with clinicopathological characteristics in Luminal B breast cancers. TRPM7 methylation was negatively associated with lymph node metastasis and radiotherapy, while positively associated with endocrine therapy in Luminal B breast cancers. [file 12885_2022_10038_MOESM4_ESM.jpg]

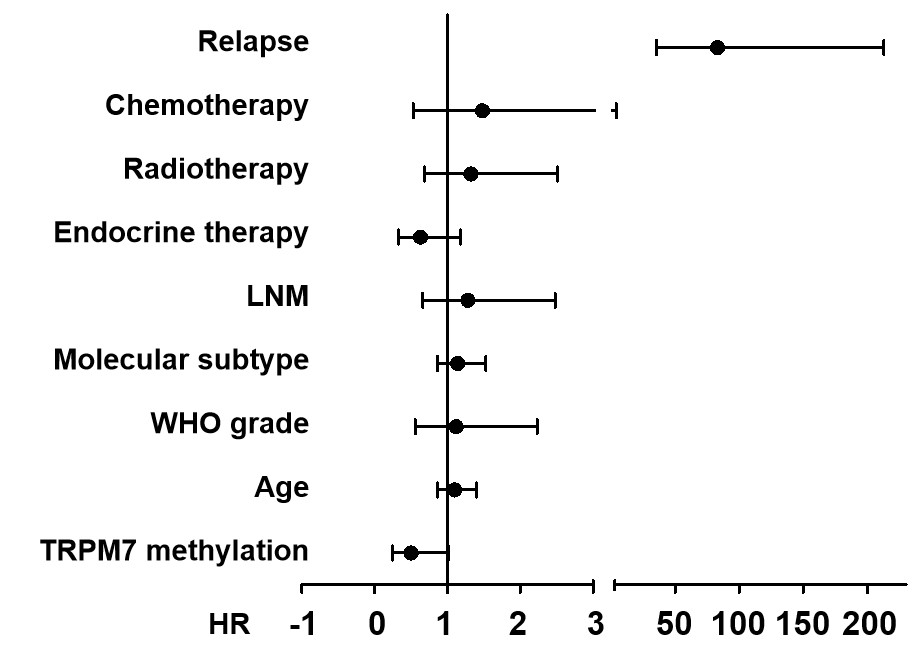

Supplement: Supplementary file 5 — Additional file 5: Figure S5. Prognostic value of clinicopathological factors and TRPM7 methylation using univariate Cox regression analysis in breast cancers. TRPM7 methylation was a potential predictor of better survival for the whole cohort breast cancer patients. [file 12885_2022_10038_MOESM5_ESM.jpg]

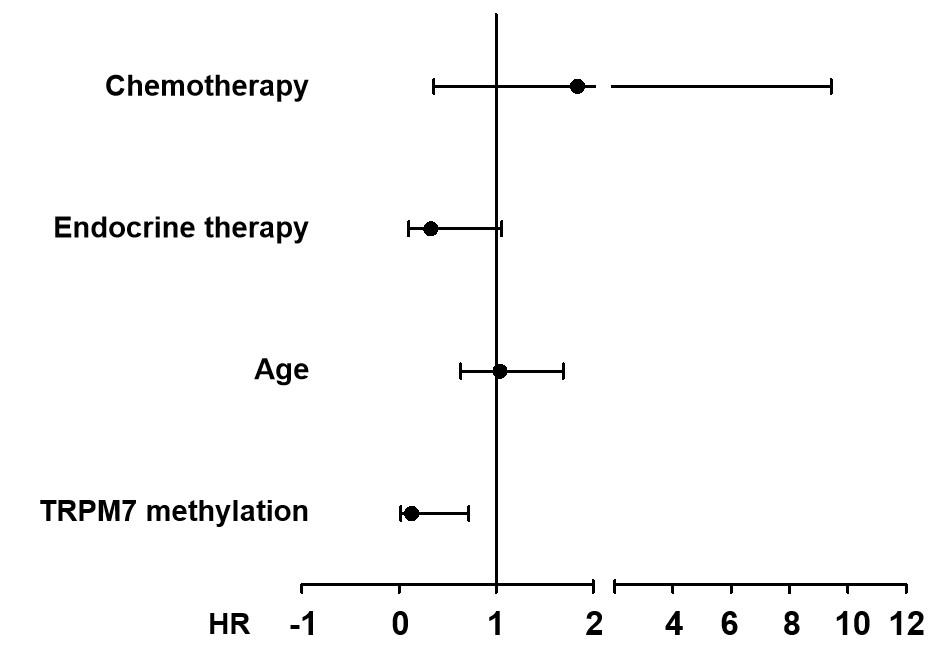

Supplement: Supplementary file 6 — Additional file 6: Figure S6. Prognostic value of clinicopathological factors and TRPM7 methylation using multivariate Cox regression analysis in Luminal A breast cancers. Cox multivariate regression showed that TRPM7 methylation and endocrine therapy is a predictor of better survival in Lumina A patients as an independently variable with respect to the age, LNM, radiotherapy and chemotherapy. [file 12885_2022_10038_MOESM6_ESM.jpg]
